# Digoxin Toxicity in a Patient with Pacemaker: A Case Report

**DOI:** 10.7759/cureus.6056

**Published:** 2019-11-02

**Authors:** Rachel E Bridwell, Keith A Baker, Christopher O Hoyte, Patrick C Ng

**Affiliations:** 1 Emergency Medicine, Brooke Army Medical Center, Fort Sam Houston, USA; 2 Emergency Medicine, St. Luke's University Hospital, Bethlehem, USA; 3 Toxicology, Denver Health, Denver, USA; 4 Emergency Medicine, Brooke Army Medical Center, San Antonio, USA

**Keywords:** digoxin, digoxin specific antibody, medication error

## Abstract

Digoxin is a cardiac myocyte sodium/potassium ATPase inhibitor with a narrow therapeutic index used to treat patients with conditions such as heart failure with reduced ejection fraction and atrial fibrillation. Currently, digoxin-specific antibody fragments serve as a therapeutic option in patients with digoxin toxicity; however, the indications for digoxin-specific antibody fragments are inconsistent, and some sources report a serum digoxin concentration of >12 ng/mL as a treatment indication. We discuss a case of an asymptomatic elevated digoxin level of 13.5 ng/mL secondary to a dosing error, who was managed without digoxin-specific antibody fragments as well as a brief retrospective chart review for patients with a pacemaker presenting with a high digoxin concentration managed with and without digoxin-specific antibody fragments, with equivocal findings.

## Introduction

Digoxin is used to treat patients with conditions such as heart failure with reduced ejection fraction and atrial fibrillation [1-2]. Digoxin increases intracellular calcium, resulting in increased contractility [1]. A therapeutic concentration of digoxin is reported as 0.8-2.0 ng/mL [3-4]. Because of its narrow therapeutic index, patients on digoxin are at risk for toxicity, which can manifest with nausea, vomiting, visual changes, altered mental status, hyperkalemia, and cardiovascular collapse [1-4]. However, the clinical significance of digoxin levels in a patient with a pacemaker is currently unclear.

Despite the declining use of digoxin, there is a high rate of toxicity in patients that are on it [5-6]. Digoxin-specific antibody fragments serve as a therapeutic option in patients with digoxin toxicity; however, the indications for digoxin-specific antibody fragments are inconsistent. According to the package insert, indications for the use of digoxin-specific antibody fragments include: ingestion of 10 mg or more in adults, 4 mg or more in children, or ingestions causing a steady-state concentration of 10 ng/mL, or in chronic ingestions, digoxin concentrations exceeding 6 ng/mL in adults or 4 ng/mL in children (FDA). Others report a serum digoxin concentration of >12 ng/mL or >15 ng/mL at any time as treatment indications [3,7]. In a review of the literature, Lloyd et al., in 2014, reported the efficacy of digoxin-specific antibodies as ranging from 50%-90%.

## Case presentation

A 75-year-old woman presented to a local emergency department with a chief complaint of lip swelling, which had resolved prior to evaluation without any interventions. She had a recent hospitalization a month prior to presentation and was treated for heart failure. Her medication list revealed that she had been discharged on digoxin. Her past medical history was pertinent for heart failure with a reduced injection fraction with a ventricular pacemaker in place.

She presented with mild chest pain. Initial vitals included blood pressure 98/28 mmHg, heart rate 104 beats per minute, respiratory rate 18 breaths per minute, and oxygen saturation of 94% on 3 L/min of oxygen via a nasal cannula. Exam revealed a 2/6 systolic murmur, a pacing device in the chest wall, dry mucous membranes, and disorientation to place and situation, which was reported to be her baseline mental status per her family members.

**Figure 1:**
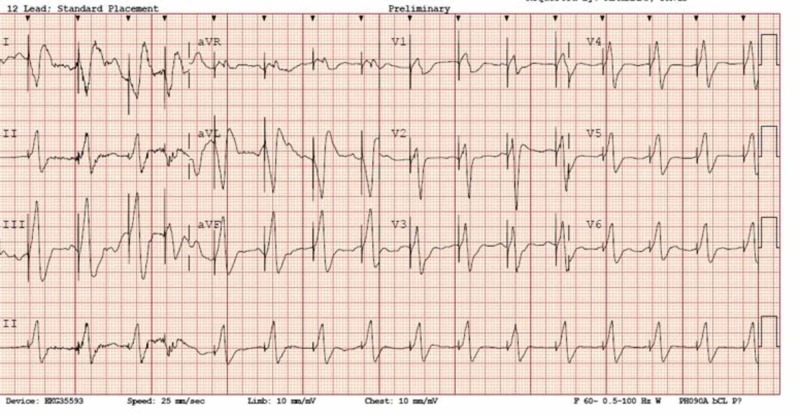
Presenting EKG showing a ventricularly paced rhythm with capture EKG: electrocardiogram

**Figure 2:**
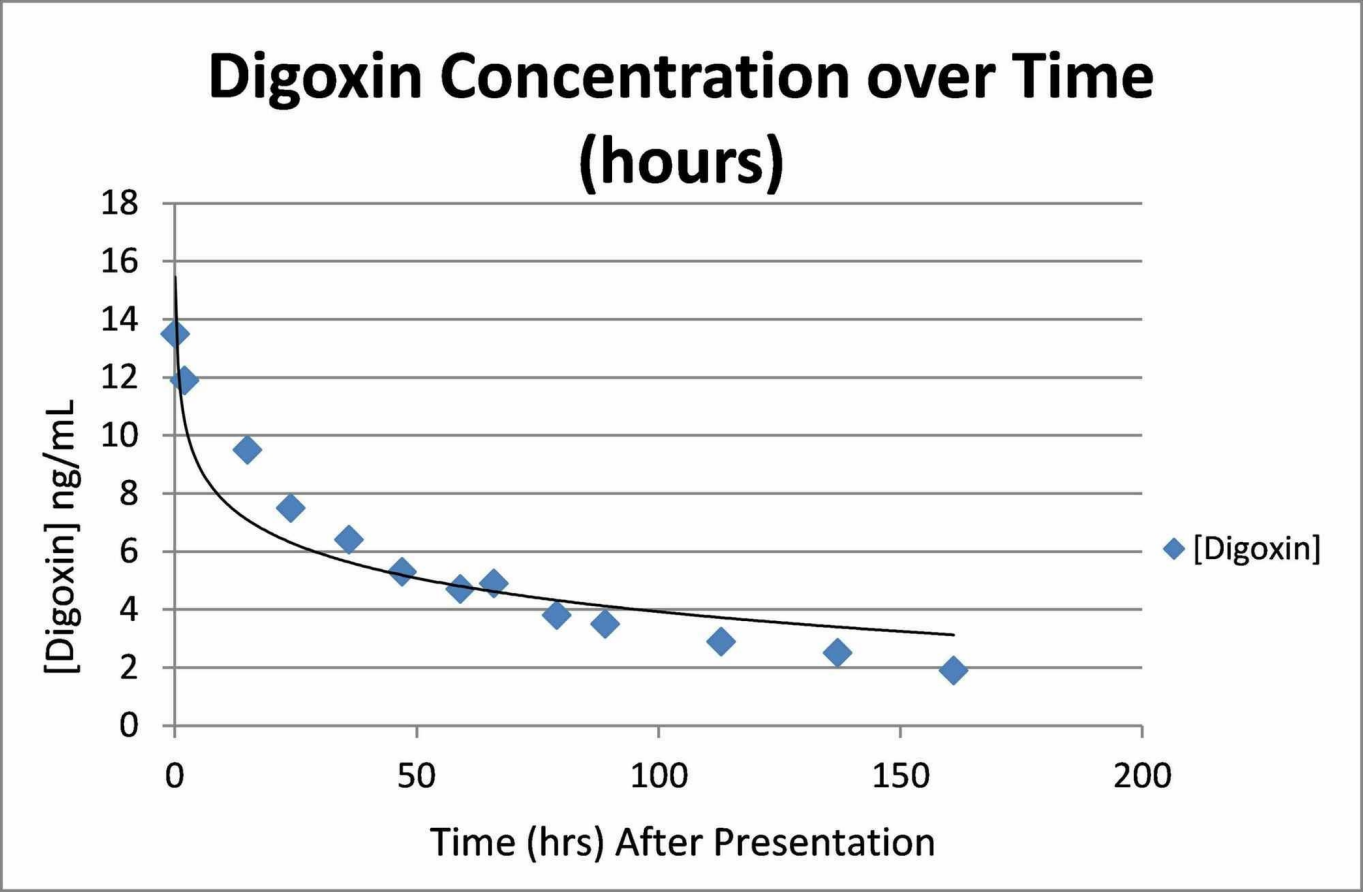
Digoxin concentration over time demonstrating normal clearance of digoxin.

EKG showed a ventricular paced rhythm at a rate of 96 (Figure 1). Lab results included potassium 4.8 mmol/L (normal range 3.5-5.- mmol/L), creatinine 1.2 mg/dL (normal range 0.7-1.3 mg/dL), troponin 0.08 ng/mL (normal <0.03 ng/mL), and digoxin 13.5 ng/mL (therapeutic window 0.8-2.0 ng/mL). After a discussion with the family and patient, the decision was made to treat the patient with supportive care in the emergency department (ED). After initial management, she was admitted and remained asymptomatic during her hospital stay. Her digoxin concentration trended down at the expected rate (Figure 2). It was recognized that the patient had mistakenly been taking a 10-fold overdose of digoxin daily since she had filled her prescription (6.25 mg daily vs 0.625 mg daily). She was discharged on hospital Day 6 in good condition.

## Discussion

We report a patient with a digoxin concentration of 13.5 ng/mL that was treated without digoxin-specific antibody fragments. Digoxin is a substrate for P-glycoprotein [8]. The potential for toxicity, coupled with its narrow therapeutic window, reinforces the importance of proper digoxin dosing and therapeutic drug monitoring [9]. The reported volume of distribution of digoxin is 5-7 L/kg. After oral administration, digoxin is absorbed and distributed in the body, reflecting a two-compartment model [8-10]. In therapeutic dosing, digoxin has an elimination half-life of approximately 36 hours in a patient with normal renal function [10]. Concentrations over the patient’s hospital stay were plotted (Figure 2), resembling first-order kinetics. The elimination half-life of digoxin in this patient was 36-37 hours, which is similar to other reports of digoxin pharmacokinetics [8-10].

The clinical significance of a patient on digoxin that has an indwelling pacemaker is unclear. Taboulet et al. reported 51 patients with cardiac glycoside intoxication managed in an intensive care unit with cardiac pacing and/or Fab fragments. Overall mortality was 13%, and they found that the prevention of life-threatening arrhythmia failed in 8% of cases with Fab and in 23% with pacing [10]. The authors encouraged the use of Fab fragments as first-line therapy. In regards to pacemakers, it can be considered that the device is protective in digoxin toxicity in that it provides a paced rhythm, thus decreasing the chances for a malignant rhythm to develop. However, it must also be considered that in a patient with digoxin toxicity, the heart is susceptible to dysrhythmia and exogenous electrical signals may precipitate a deleterious rhythm as suggested by Taboulet et al. Our patient did not develop any dysrhythmias and her repeat EKGs revealed that she remained in a ventricularly paced rhythm throughout her stay.

## Conclusions

An elevated serum digoxin concentration can serve as an indication for treatment with digoxin-specific antibody fragments; however, the treatment indications for digoxin-specific antibody fragments are not consistent. The effects of an indwelling pacemaker in a patient with digoxin toxicity are uncertain. These should be considered in the initial management of digoxin overdose in the emergency department.

## References

[1] Aravanis C, Michaelides G (1959). Paroxysmal auricular flutter and right bundle branch block following digitalis therapy. Am J Cardiol.

[2] Morris SA, Hatcher HF, Reddy DK (2006). Digoxin therapy for heart failure: an update. Am Fam Physician.

[3] Pincus M (2016). Management of digoxin toxicity. Aust Prescr.

[4] Haynes K, Heitjan D, Kanetsky P, Hennessy S (2008). Declining public health burden of digoxin toxicity from 1991 to 2004. Clin Pharmacol.

[5] Yancy CW, Jessup M, Bozkurt B (2013). American College of Cardiology Foundation. American Heart Association Task Force on Practice Guidelines. 2013 ACCF/AHA guideline for the management of heart failure: a report of the American College of Cardiology Foundation/American Heart Association Task Force on practice guidelines. J Am Coll Cardiol.

[6] Lloyd M (2014). Digoxin-specific antibody fragments in the treatment of digoxin toxicity. J Emerg Med.

[7] Oncu S, Gelal A, Aslan O, Ucku RS (2018). Appropriateness of digoxin measurement in hospitalized patients. Biochem Med.

[8] Ziff OJ, Kotecha D (2016). Digoxin: the good and the bad. Trends Cardiovasc Med.

[9] Hastreiter AR, John EG, van der Horst RL (1988). Digitalis, digitalis antibodies, digitalis-like immunoreactive substances, and sodium homeostasis: a review. Clin Perinatol.

[10] Taboulet P, Baud FJ, Bismuth C, Vicaut E (1993). Acute digitalis intoxication — is pacing still appropriate. J Toxicol Clin Toxicol.

